# Sport for Development and COVID-19: Responding to Change and Participant Needs

**DOI:** 10.3389/fspor.2020.590151

**Published:** 2020-12-23

**Authors:** Marlene A. Dixon, Ashlyn Hardie, Stacy M. Warner, Emmaculate Awour Owiro, Dennis Orek

**Affiliations:** ^1^Department of Health and Kinesiology, Texas A&M University, College Station, TX, United States; ^2^Department of Kinesiology, East Carolina University, Greenville, NC, United States; ^3^Independent Researcher, Nairobi, Kenya

**Keywords:** sport for development, hybrid organizations, case study, girls sport, lifecourse theory

## Abstract

As COVID-19 hit in the Spring of 2020, substantial challenges began to emerge for individuals around the world. In this empirical piece we examine the impacts of COVID-19 in the sport for development (SFD) context, as it relates to the individual participant, as well as how those individual needs impact the organization itself. This case study, explores a hybrid SFD organization, Highway of Hope (HOH) in Kenya, and the actions of local leadership in response to emergent participant needs during the onset of the pandemic. Using a case study approach, involving journal responses from program youth participants and local program leadership, along with other field and meeting notes, themes were generated to highlight the most salient challenges and experiences faced by individual participants, as well as the means of addressing those challenges by HOH. Participant journals consisted of both positive and negative thematic findings. Positive experiences included Family Time, Rest, and Practicing Better Hygiene, while negative experiences revolved around Restriction of Activity, and Difficulty at Home. The overall impact on participant lives were expressed in themes such as Socio-emotional, Physical, and Sport-Specific. Further, practical implications for grassroots and hybrid SFD programs during times of unprecedented challenge and notable turning points are highlighted.

## Introduction

*When the first COVID-19 case was confirmed and announced, there was evident panic and fear that engulfed the whole crowd at Upperhill school where the games were being hosted. That one announcement changed a lot of things, within a few minutes people started to treat each other as strangers, there were quick and regular visits to the open public water points (taps) to hand-wash, and people began self-distancing. On the same day as Highway of Hope (HOH) girls were playing their final game, the government, through the ministry of education and ministry of sports issued a directive to close all public events and sports around Nairobi. That was the beginning of a new normal in my life. (HOH Kenyan Program Director)*.

During the Spring of 2020, the COVID-19 pandemic wreaked havoc on everyday life for over 7.5 Billion people around the globe. While the initial declaration of the outbreak was announced in January, it was not until March that regulations on social distancing, group gatherings, business operations, and various facility closures (including sport training facilities) were put into place (Zinner et al., 2020). Within the sport sector, the closing of facilities, events, businesses, tournaments, and previously normal operations lead to a freeze on a multibillion-dollar global industry (Robert Pearl, 2020). While this freeze was a global adjustment for the sport community, contextual factors such as degrees of lockdown, community support of safety measures, and specific restrictions placed on sport were major determinants of differences in sport participant experiences (Kelly et al., 2020). More specifically with regard to youth sport, public health professionals have seen increased childhood obesity in the months following the virus outbreak, and they predict will continue throughout the duration of limited sport and play model (Robert Pearl, 2020). Health experts have also found that these physical health issues in combination with social isolation have already, and will continue to be a serious issue of adolescent mental health for the foreseeable future (Robert Pearl, 2020).

Acknowledging these challenges, Warner and Martin (2020) also wrote about the impact the COVID-19 pandemic has had on sports worldwide. However, they argued that while the pandemic has presented enormous challenges, it has also created opportunities for growth and change within sport organizations. Warner and Martin (2020) stated:

How our sport systems react will be key to demonstrating, justifying, and legitimatizing just how fundamental and important sport is to individuals' overall well-being, health, and everyday life. Thus, the COVID-19 crisis can and should be viewed as an opportunity for sport fans, athletes, and sport managers to validate the role and power of sport to positively impact society.

Although their whitepaper highlighted various sport systems and organizations (e.g., U.S. youth sport, NBA, MLB, etc.), it failed to consider Sport for Development (SFD) programs or organizations. SFD organizations provide sport in various forms to millions of young people around the world, and sport is often only one component of more broadly designed programs that impact both individuals and communities (Schulenkorf et al., 2016).

The SFD domain is ever changing to become more intentional in mobilizing grassroots projects and empowering local leadership. However, many of these organizations represent the interests of a variety of stakeholders that inform, assist, and sometimes hinder integrity of grassroots providers' vision and implementation of SFD programming (e.g., Giulianotti et al., 2016; Svensson, 2017; Dixon and Svensson, 2019). The ability of organizations to adapt to changing environmental conditions and stakeholder input remains a concern for the long-term development and sustainability of SFD organizations (Dixon and Svensson, 2019). We argue that this global pandemic, while challenging, may provide multiple opportunities for SFD programs. Specifically, it could demonstrate the role SFD can play in addressing the most salient participant needs, and how SFD organizations adapt in operations and direction to meet emerging participant needs.

Using a life course perspective, COVID-19 could be viewed as what Giele and Elder (1998) call a “turning point.” From an individual perspective, a turning point is a moment when a decision must be made between two life choices—for example, a choice based on a job offer, a geographic location, or the choice to marry/not, or to have children/not. Dixon and Svensson (2019) demonstrated that SFD organizations encounter turning points in their life course where disruptions (e.g., the introduction of stakeholders, economic needs, and/or changes in organizational leadership) create opportunities for organizations to adapt and adjust to remain both viable and true to the organizational mission. Changes can include adaptations to structure, content, programming, relationships, financial partnerships, and stakeholders (Dixon and Svensson, 2019). Leveraging this concept of turning points and the opportunities they can create, the purpose of this study is to better understand the voices and perspective of grassroots SFD participants during the COVID-19 crisis. The ultimate goal is to learn how one hybrid SFD organization responded to the changing needs of its participants, and draw from that learning toward other SFD organizations.

### Adaptability Within SFD Organizations

New SFD initiatives are difficult not only to build, but also to sustain because they often involve scarce resources, as well as multiple stakeholders, agendas, and organizational forms (Giulianotti et al., 2016; Svensson, 2017; Svensson and Seifried, 2017; Dixon and Svensson, 2019). Several scholars have noted the complexities of developing and operating SFD programs as well as the multiplicity of stakeholders that can be involved, including non-profits, corporations, inter-governmental agencies, governments, and high-performance sport organizations (Svensson, 2017; Svensson and Seifried, 2017; Dixon and Svensson, 2019). One way that organizations can manage multiple stakeholders and interests is to adopt a hybridized form, embracing structures and processes that combine multiple organizational logics and embrace the input and values of multiple stakeholders (Battilana et al., 2014). Organizational logics are socially constructed frames of reference from which individuals derive meaning and function within their organizations (Thornton and Ocasio, 1999; Battilana et al., 2014).

The process of hybridization and functioning within hybrid forms is not always smooth, often bringing about organizational tensions (Smith and Lewis, 2011). In some ways, hybrids also can be slow to adapt because of the need to address multiple stakeholders and interests, and the need to resolve tensions before moving forward. However, others have suggested that hybrid organizations have superior flexibility in the face of environmental challenges because of their multiple sources of resources, their flexible structure, and their malleable functions (Svensson and Seifried, 2017; Dixon and Svensson, 2019).

Hybrid organizations, therefore, are an excellent context to study organizational adaptations (Svensson and Seifried, 2017; Dixon and Svensson, 2019). For example, Dixon and Svensson (2019) described the evolution of a hybrid organization from conception through the first 4 years of operations, highlighting both the tensions and the opportunities created from the combination of organizational partners and multiple stakeholders. In their study, like in the case of many SFD organizations, the impact of multiple stakeholders (especially with heavy influence from Global North partners) can limit the flexibility of grassroots sport organizations to respond to emergent need in their own contexts and constrain choices for such response (Schulenkorf, 2012). As Svensson (2017) pointed out, grassroots entities can face considerable pressures regarding the funding, management, program design, areas of focus, and program outcomes by external stakeholders. For one example, program curriculum that is developed from Global North partners may not be sensitive to or capable of fulfilling cultural needs within a context, especially if those needs are in flux (Schulenkorf, 2010). Yet, grassroots partners may adopt the curriculum anyway. In this and other ways, they may be unwilling to set strong organizational boundaries, especially if they threaten valuable resources the organization needs for survival (Svensson, 2017).

Although hybrid SFD organizations face challenges, within a culture of trust, cultural sensitivity, and openness to new ideas, organizational leaders, particularly in hybrid organizations, have been able to create new ways of responding to organizational problems (Smith et al., 2012; Doherty et al., 2014; Dixon and Svensson, 2019). This process of negotiating tensions and thinking beyond current operational or structural constraints can build capacity of the individual organizational members, as well as the organization as a whole (Doherty et al., 2014; Dixon and Svensson, 2019).

With the onset of COVID-19, organizations were forced to re-evaluate participant needs, and plans for meeting those needs (*cf*. Warner and Martin, 2020). Through viewing COVID-19 as a turning point with opportunities and constraints for grassroots hybrid SFD organizations, we can further our understanding of the lived experiences of SFD participants. Ultimately, the insights gained can help shape the future for SFD organizations (Battilana et al., 2014; Smith and Besharov, 2017). Thus, the current study addresses the following research questions:

1. What were the salient changes in participants' lives created by the onset of COVID-19 and how did those changes impact participant lives?
2. What were the emergent participant needs, and in what ways did the SFD organization adapt to meet the emerging participant needs?
3. How might those changes affect the future of the organization?

## Method

### Case Setting

Given the goal to represent the lived experiences of SFD grassroots participants, a qualitative descriptive case study approach, with its focus on naturalistic inquiry, proved the best methodological fit (Eisenhardt, 1989; Sandelowski, 2000; Creswell, 2013). A number of SFD scholars have argued that research should be constructed not only to highlight local voices, but also to engage local collaborators in the study design, questions, and methodology that inform and undergird the inquiry (e.g., Giulianotti et al., 2016; Schulenkorf et al., 2016; Darnell et al., 2018). Therefore, the study design takes a collaborative approach, whereby every step of the study was co-created, analyzed, and authored by the U.S. and Kenyan partners.

### SFD Grassroot Hybrid Organization

Highway of Hope (HOH) operates in Nairobi, Kenya in the informal settlement of Kibera. It exists as a partnership between local community members and several U.S. partners who help design, implement, and refine the program. Prior to COVID-19, the program had grown from one team of 12 adolescent girls from one school in Kibera, to 5 teams across 3 schools, with 59 young women taking part in the sport and mentoring activities. Pre-COVID-19, the programming consisted of basketball training 5 days/week for each team, and weekly mentoring sessions. The teams participated in their school-based basketball leagues, typically playing several games per week, until these were abruptly suspended in early March.

The program (co-created and supported by Kenyan and U.S. partners) was delivered by local coaches and mentors. These coaches and mentors had relationships with the participants that spanned several months to 3 years. The U.S. partners have been involved with the project for since its inception, spending extensive time on-site with the participants, coaches, mentors, and program leaders. No site visits were conducted during the time of this study (March 2020- August 2020).

From April 2020 to date, the program decreased from 59 girls in 3 schools to 16 young women from one school, based on physical space capacity of the organization. As of mid-May, these young women participated in academic tutoring and mentoring three times a week in a safe environment that allowed for social distancing. Initially, all sport training was discontinued, but as time passed, some restrictions were lifted, and the team could practice 1–2 times per week in small groups. They could eventually participate in some 1-h scrimmages, but the courts were limited to only 15 players at a time. The participants also took part in individual physical training, but did not have access to any basketballs, goals, or sport equipment for basketball skill development.

### Data Collection

Case studies rely on a variety of data collection methods in order to understand the case in as much depth as possible. These collection methods can include archives, observations, field notes, journals, and interviews (Eisenhardt, 1989). The data from this study are part of a larger ongoing project involving several of the research team members (for more context and information about the organization see Dixon and Svensson, 2019). However, for this study, a specific emphasis was placed on a journaling activity related to COVID-19. This study received all necessary human subjects' approvals.

### Observations/Field Notes

Observations and field notes of the organization for this study included regular online meetings of project sub-groups, bi-monthly conference calls involving the entire project team, weekly informal interactions with project team members, participants, and program leaders via zoom, WhatsApp, and email. During these interactions, extensive field notes were kept (Kieren and Munro, 1985). Meetings and conversation focused largely on the changing landscape of the COVID-19 situation, the changes in program delivery, and the need for reducing the scale of operations. These informal conversations were documented in the same manner as field notes, with some notations taken as jottings or key words, and others as verbatim quotes (Emerson et al., 1995).

These conversations and observations were organic to the operations of the organization and were not specifically derived around life course or organizational hybridity theories. They were the practical, emergent conversations within the organization as they navigated the COVID-19 pandemic. Thus, while specific questions relative to life course theory and/or hybridity tensions/opportunities were not explicitly utilized in the conversation, the conversations themselves naturally lent themselves to both of these perspectives.

### Participant Journals

In addition to the ongoing conversations with the program leaders, coaches, and mentors, the program participants engaged in a targeted journaling exercise specifically designed to understand their COVID-19 experiences from a life course perspective (Giele and Elder, 1998). Specifically, one element of life course theory is the identification of turning points and the specific contexts and outcomes surrounding those turning points (e.g., what happened, who was involved, what was the outcome?). Based in this concept, and previous sport management literature using life course theory (see Bruening and Dixon, 2008; Dixon et al., 2008), the journaling activity consisted of nine questions, derived from the entire project team (the U.S. researchers and Kenyan program implementers and with input from several of the participants), regarding the changes they experienced as a result of COVID-19. This collaborative approach helped ensure that the journaling prompts were simultaneously consistent with life course theory and relevant to the context. Example questions included: How has COVID-19 impacted your life? What are the biggest changes to you? How has COVID-19 impacted your ability to practice and play basketball? The 16 participants (See Table 1) were instructed to give some general insights about these experiences and to provide specific examples. Mentors debriefed the journaling activities with the small group of participants to help them process any issues that were troubling or needed further action. These documents –observational field notes, journal entries, interviews/conversations, and journals—formed the in-depth “story” of the organization, which is the basis of analysis (Eisenhardt, 1989; Giele and Elder, 1998).

**Table 1:** Participant pseudonyms and demographics.

**Pseudonym**	**Age**	**Year in School/Program role**
Jennifer	16	^**^Form 2
Sophia	14	Form 2
Sarah	16	Form 3
Serenity	16	Form 2
Vanessa	17	Form 3
Melissa	15	Form 2
Theresa	17	Form 3
Priscilla	14	Form 2
Grace	15	Form 2
Samara	15	Form 2
Diana	18	Form 4
Savannah	16	Form 2
Victoria	17	Form 3
Maya	18	Form 3
Mila	16	Form 2
Miriam	17	Form 3
Marlene	N/A	U.S. Program Director
Irene	N/A	Kenyan Program Director
Dennis	N/A	Head Basketball Coach
Emmaculata	N/A	Head Mentor

** *In the Kenyan school system, children typically attend 8 years of lower school, namely grades 1–8. Then, upper or high school consists of 4 years, Forms 1–4*.

### Data Analysis

Using qualitative content analysis, we analyzed the data according to the research questions, with the purpose of creating a “comprehensive summary of the events in the everyday terms of those events” (Sandelowski, 2000, p. 336). That is, using the field notes, meeting transcripts, and the journal entries, the entire research team (both U.S. and Kenyan project partners) compiled the story of the organization as it relates to the specific purpose of this study. For Research Question 1 the data were drawn almost exclusively from the participant journals. For Research Questions 2 and 3 data were derived and interpreted from participant journals, team meetings, and informal conversations/field notes.

We employed an inductive process for developing codes and themes. This required a multi-step process and attention to what each participant had to say, rather than trying to force data into pre-existing codes (Miles et al., 2013). In the first step, Kenyan team leaders read through the documents and journals and provided feedback to the research team as to what elements of the story they considered salient and how they interpreted that information. This process helped accomplish a goal of hearing from the grassroots providers themselves, as well as having the analysis rooted in local understanding and interpretations of participant voices, rather than relying solely on foreign, researcher interpretations (see Giulianotti et al., 2016; Schulenkorf et al., 2016).

Next, the entire research team read through the data, and made notes of overall comments related to the main research questions and with specific mindfulness to turning points in individual lives and the organization itself (participant experiences and responses, organizational responses, and future directions). In the second step, we developed pattern coding, which pulled the overall first-level comments into groups or preliminary themes. Essentially, the goal in this step was to identify patterns and key excerpts from the data (representative themes) that described the overall essence of what the participants collectively communicated through their journals, interviews, or field notes (Miles et al., 2013).

**Figure 1:**
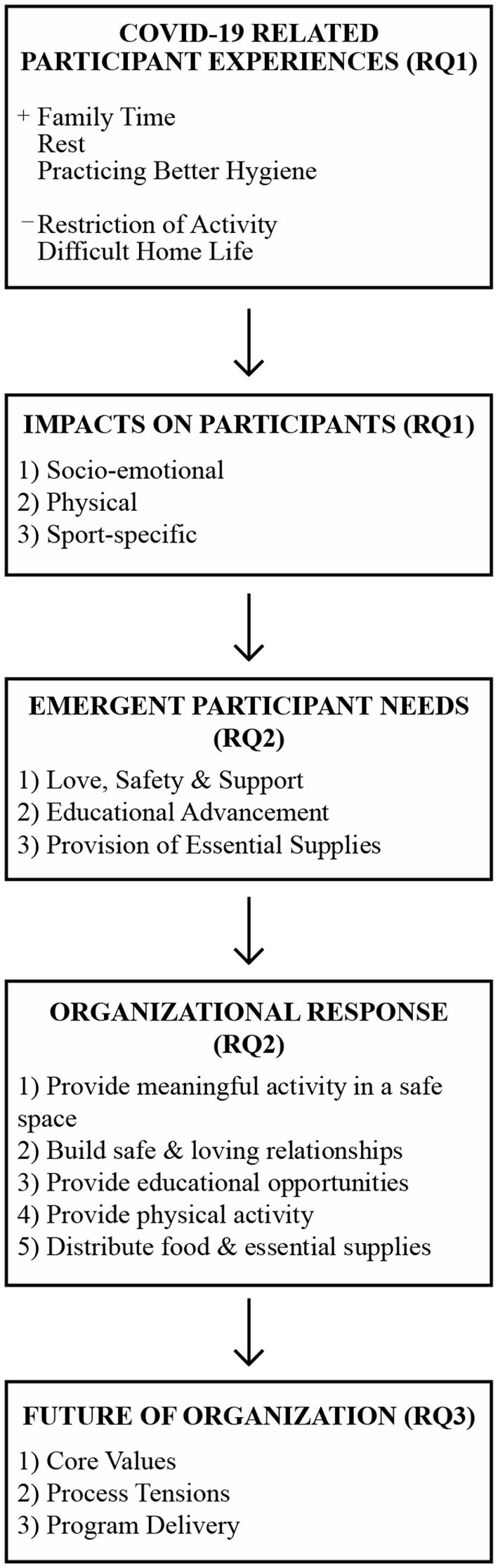
Visual summary of results.

Then, the research team generated themes using the patterned codes as well as the initial interpretive feedback from the Kenyan project leaders. Thus, the theme generating process went beyond simply finding commonalities between meaning units and grouping them together. In generating themes, we collectively discussed and made evaluative decisions about what information was most relevant to each research question, and we interpreted salient portions of the journals, field notes, and interviews at a low-inference level (Sandelowski, 2000, 2010). Finally, we located representative quotes from the data to support these interpretations, being careful to stay true to participant description of their own lived experiences (Sandelowski, 2000, 2010). Figure 1 visually illustrates the emergent themes as presented in the results below.

## Results

The results are presented by research question and while not all the participants' views can be represented the most salient ones are provided. The results of RQ1, “What were the salient changes in participants' lives created by the onset of COVID-19 and how did those changes impact participant lives?,” revealed the COVID-19 impact on the lives of the young participants in the SDP program. While COVID-19 has presented a number of challenges for individuals around the globe, there have been emergent opportunities for positive takeaways, as well. The positive changes (*Family Time, Rest, and Increased Attention to Sanitation and Hygiene*) will be first be presented followed by the challenges (*Restriction of Activity and Difficult Home Life*). After this, the impact on participant lives (*Socio-emotional, Physical*, and *Sport-specific)* will be presented.

### Positive Experiences

Some of the positives that have come out of the pandemic for the participants are *Family Time, Rest, and Practicing Better Hygiene*. First, the participants expressed that they were able to spend more time with their families while sporting activities are not taking place. Serenity explained, “I have never had time to spend with my family before, but right now I am indoors with them and we share bright moments.” The pandemic highlighted that the time spent on sport is often at the expense of time with one's family. As Sarah explained, “I used to spend most of my time in school and basketball but now I have the whole day with my family.” Melissa added, “Now I also finally have time to spend with my family. Unlike before, when I was tangled up in sports.” Thus, increased *Family Time*, was a theme that emerged from the data and seemed to be a positive for the SDP participants.

*Rest* was the second theme to emerge. Several players who needed personal time or players that have been battling an injury mentioned Rest as a positive aspect of COVID-19. For example, Priscilla said, “Positively it has given me a lot of time to rest.” While Theresa wrote, “My mental health is not at all that disturbed because I have injuries that actually have more than enough time to recover.” The time away from basketball has given some players the opportunity to try out other activities apart from sport and discover new interest. As Savannah explained, “COVID-19 has given me a break to recover from injuries and discover new hobbies.” Unexpectedly, the pandemic provided a time for a break, which allowed the participants to rest, recover, and discover new interests.

Finally, the participants and program leaders noted that the community in general had adopted a higher standard of hygiene. This theme entitled, *Practicing Better Hygiene*, was noted as being especially important in areas like the slums where sanitation is already a prominent issue. As Melissa said, “On the positive side, it has helped many of us to maintain cleanness.” From this perspective, the program leaders suggested this may increase the health and well-being of the community overall. However, the participants also expressed the weariness and annoyance of constantly managing this aspect of life. As Sarah expressed, “Now we have to sanitize everywhere ever time, which is a bit tiresome.” Sanitation and precautionary practices like wearing a mask, hand washing, and social distancing were all also new concepts for the adolescents to adjust to and take responsibility for.

### Challenges

While it is encouraging to learn of the positive takeaways the participants have drawn from this experience, the challenges that COVID-19 has presented remain. Overall, the negative experience of COVID-19 stems around *Restriction of Activity* exacerbated by a *Difficult Home Life*. Our findings showed frustration with the restrictions placed on the community due to government lockdowns and curfews, as well as the closing of schools and other public places. Because most of the participants do not have access to online classes, participants felt that the pandemic has especially negatively impacted them.

### Restriction of Activity

The *Restriction of Activity* theme was comprised of the participants' discussions surrounding the closing of schools, canceling of sport, and curfews. The detachment from their teachers and support group from school was noted by the majority of the students. The schools also offer resources like books and sports equipment and thus the detachment from both social and physical resources was especially noteworthy and a negative. Mila stated, “From the time that school was closed I really missed out on so many things, about school, family, sports, and even friends. I miss my teachers and classmates.” It was clear the social support found through school was missing, as well as the resources. Grace added, “It makes one feel uncomfortable, especially students, they are supposed to learn through the internet and television of which not all students can afford those materials.”

This closing of the schools consequently impacted sport opportunities for the participants. As Vanessa explained:

COVID-19 caused all the schools to be closed. Since the schools were closed, there are no open courts because the courts are all in the school. So now we no longer train because we are not allowed to enter the school premises and there are no public courts.

Victoria added, “Having no school has really impacted my access in sports since I have no access in basketball courts. I also cannot be with my coaches, and teammates.”

The cancellation of sport programming was devastating to many as basketball was not simply an after-school activity, but also part of a larger social community. As Sarah explained:

Before the pandemic began we used to go to tournaments, train until late hours, and workout happily with teammates but right now all the courts have been closed, we are not supposed to hang out and hug our teammates as we did before.

Grace's comments also reflect those of many others. She said, “COVID-19 impacted the closing of schools, which led to no accessibility of basketball courts, training facilities, and coaches. This has led to the slow decline of my basketball skills and abilities as a player.” Victoria added, “COVID-19 has affected me mentally and it is because I miss training with my teammates and going to school.”

### Difficult Home Life

In addition to these external restrictions the home lives of the participants were difficult because of parent unemployment, which increased the stress on the participants. Savannah said, “COVID-19 has lead to the closure of schools and loss of jobs too. This pandemic has brought about budget cuts in the household and also idleness of children at home.” Sarah added, “COVID-19 has brought about many needs in our lives that we need. Everyone has been forced to stay home, and unemployment increased. There is a need for food and shelter which are the main things. The food prices have also raised.”

Life at home was also unpleasant because of few resources in the community, leading to boredom. The theme of *Difficult Home Life* is also reflected in numerous quotes from the participants about how bored, lonely, and tiresome their lives are, especially as they consist of home chores and tutoring. Consider the following quotes from the participants:

I feel so bad and embarrassed by this disease because all my time I am at home doing nothing after my house chores. (Samara)

Due to the pandemic, everybody in our family is now at home starting from my parents to my brothers and sisters. In my family, since COVID-19 started we have been just staying at the home with my family with nothing to do at all. The only thing we have been doing is just to eat and read with each other. (Victoria)

COVID-19 has impacted my relationship with my family members by avoiding talking to them. Before we used to meet and socialize together, I have missed them and would like to see them and talk to them. (Priscilla).

The participants experience of COVID-19 revolved around severe *Restriction of Activity* in addition to their *Difficult Home Lives*. COVID-19 was clearly an unwelcome change in their lives.

### Impact on Participant Lives

After describing their experiences, the participants went further to discuss the impact that the disruptions related to COVID-19 had on their lives. These impacts, both in and out of sport were profound. The participants expressed impacts on multiple life areas including *Socio-emotional, Physical*, and *Sport-specific*.

### Socio-Emotional

Socio-emotional impacts include the feelings of sadness, loneliness, and disappointment that the girls are experiencing as a result of not being able to play, and interact with their friends, teammates, and supporting network program coaches and mentors, but also not being able to hug, share physical contact with those same individuals. The following three quotes illustrate this theme:

COVID-19 has affected my mental health, especially quarantine. It has affected my usual activities. My level of loneliness, and feeling alienated has also gone up. This has saddened me a lot, but things will get better soon. (Melissa)

So many problems have been disturbing my mind, like peer pressure. COVID-19 has affected me mentally and it is because I miss training with my teammates and going to school. (Jennifer)

For my mental health, it has made me to be emotionally unstable, since I can no longer be with my fellow teammates, coaches, and beloved ones. I feel depressed on what is going on and I do wish that things would go back to how they were. (Mila).

Interestingly, many of the participants expressed their feelings about the impact of COVID-19 in terms of regrets or things they took for granted prior to the regulations. As Savannah stated, “This makes me feel regretful of the time I wasted when the changes I left to go by. I really miss getting out of the house for tournaments.” Sarah added, “Life before COVID-19 pandemic was such a blessing, that we all took for granted.” In particular, the participants missed basketball and their teammates. As one example, Veronica said, “What I took for granted was that I did not think that my teammates will 1 day be separated from me.” And Melissa said, “I miss my coaches, my teammates, and even my opponents since we can no longer meet and have fun together and we cannot plan and train together.”

### Physical

COVID-19 also impacted physical aspects of the participants lives. In fact, every participant commented on the impact of COVID-19 on their fitness levels and physical well-being. These are illustrated in the following quotes:

I used to have fun with my mates but right now we cannot meet and I really miss them. I feel so bad. I feel as if I'm growing fat on the inside. I just miss the court, the balls, our coaches, and my mates. (Theresa).

COVID has impacted my physical health by making my body weak, and impacted my mental health by being idle, hence making my thinking capacity to be low. Training exercise also improved my mental health and kept me busy. (Priscilla).

I used to have a flat belly, but now that I have to stay at home my belly is enlarging and becoming a pot. (Savannah).

### Sport-Specific

Related, the lack of basketball activity impacted participants' development of basketball skills; these are expressed in the theme *Sport-specific*. As Grace explained, “COVID-19 impacted the closing of schools, which led to no accessibility of basketball courts, training facilities, and coaches. This has led to the slow decline of my basketball skills and abilities as a player.” Vanessa added, “Personally, my skills now have deteriorated and I can't handle the ball like I used to before. I miss my shooting, like three-point shooting, and defense.” Similarly, Savannah said, “My skills in basketball are stagnant and have started to wear-out for I have no access to improve my skills.” The athletes expressed frustration at having worked very hard to gain a skill, then watching it deteriorate over time. As Priscilla said, “When this is over, I hope I don't have to start from square one.”

Overall, the participants' articulated both positive experiences (i.e., *Family, Rest*, and *Practicing Better Hygiene*) and struggles (i.e., *Restriction of Activity, Difficult Home Life*) as well as the various ways that COVID-19 impacted their lives in and out of sport. The emerging participant experiences highlighted core participant needs as well as opportunities for HOH local leadership to take on new ways of helping their participants, and the organizational adjustments it would take to do so.

### Organizational Response to Participant Needs

Research Question 2 asked, “What were the emergent participant needs, and in what ways did the SFD organization adapt to meet the emerging participant needs? The review of the participant data and daily interactions with the participants led the local program leaders to suggest that the three main needs of the participants were the following: (1) *Love, Safety, and Support*, (2) *Educational Advancement*, and (3) *Provision of Essential Supplies*. In the words of the Head Basketball Coach:

The pandemic has exposed most of the students, especially girls, to a lot of situations which has left them vulnerable to fall into wrong hands and make bad choices. The lack of safer space in the slums and support has caused a lot of pressure for the students.

Multiple youth participants expressed gratitude toward the organization in their journal entries, saying that “the organization helped [give them] mentorship classes, which helped my life because outside there are young teenagers engaged in bad company,” and “since we are on a long holiday [they] are more likely to be engaged in bad things like prostitution, getting pregnant, and stealing.”

### Love, Safety, and Support

To address the first need—*Love, Safety, and Support*—the organization arranged a safe space and began to offer small-group and individual mentorship. This has assisted the students in having time in a safe space away from slum pressure as well as positive engagement with their teammates, coaches, and mentors. As stated by the Kenyan Program Director:

The other challenge that was presented to us was to have girls' teenagers, those who did not travel to the countryside, sit idle and move around their neighborhood without any serious engagement. We have had a nationwide outcry of teenage pregnancies that is worrying during this COVID-19 period, which was documented and aired in our national televisions. This called upon the HOH team to think of a rapid response toward engaging the girls in a meaningful tutorials, training and mentorship out of the normal school program. We've not been able to meet to all the girls in the program but we are grateful for the opportunity to reach out to the small number, which has been made possible due to the long working relationship we've had with the girls and their parents. This has presented me as a leader in the program to rethink of the design as we implement this program and other similar programs in the future: Parental/guardian involvement is very key and important at the onset of every sport program targeting youths/children below 18 years in our context.

The mentoring and tutoring structure, delivery and actual content is a departure from the original HOH program design, which follows a weekly group mentoring model and pre-developed curriculum. However, based on notes from the bi-monthly team meetings, the entire program team felt the adaptation was true to the organizational mission, and presented a valuable opportunity to build deeper relationships with a smaller number of young women. The team members agreed that the socio-emotional, physical, and mental health of the girls was of primary importance. As explained by the Head Basketball Coach, “The coaches and mentors have also maintained love and support to the kids by being the go-to person whenever they have issues they would want to share.” Participant journals were full with statements like, “I would like to thank HOH for how they mentored us, because they make our future safer than those young youth idling outside and having nowhere to be helped,” “they have given me support and love, and shared everything with us,” and “they have supported me and mentored me in such a way that not to give up on life.”

As an additional measure to address a desire to have some physical activity and interactions with coaches, HOH has also been able to secure a training ground and the students get to play thrice a week which is helping them to stay active, fit, and out of any possible trouble. It is clear that providing Love, Safety, and Support are fundamental to HOH, and this was a participant need that the organization had to adapt to providing.

### Educational Advancement

Second, it became obvious that educational needs were not being met for many of the participants. As explained by the Head Mentor:

The government closed schools and most of the children could not access online classes due to lack of gadgets and Wi-Fi. This meant that most of the students were heavily disadvantaged. The coaches and mentors have now been forced to step up and be the teachers to the kids to ensure their education continues. The program has gone a long way to use the coaches and mentors to personally tutor the kids and provide them with practical job skills training that will help them in the future.

Specifically, HOH helped by setting up a study group for the students where they can come and learn together. The program implementers, of their own initiative and through their local community networks, secured a sufficiently large space for the study group. The teaching is done by the coaches and a few invited individuals who have come out to support the study group. The students also have access to a library through the partnership of several local organizations. Grace expressed her concern about home schooling by saying:

Before we learned by going to school, hence now we have to learn through the internet and television. This story makes me feel uncomfortable about learning through the internet and television because not all students can afford those materials… and the teacher may explain something that we cannot understand without asking questions.

The young women's appreciation for the HOH response to these needs was overwhelmingly positive. Journal entries included statements like those from Vanessa,

My biggest needs during this time is to really study hard so that when I go back to school I can catch up with the teachers lessons and my mind won't be shut down. HOH has really helped me because by now I would be at home and studying alone, which is boring, and they helped me by creating a study group.

From a program development perspective, the HOH program implementers took the initiative not only to secure the space, but also to design the tutoring program as well as the practical skills/leadership training component. Interestingly, the development of this practical skills/leadership component was something that was a strategic goal of the organization for 2020 and had not received much attention or traction due to the rapid growth of the program and the emphasis on basketball during the inter-school season which takes place from February to April. The reduction of the program size and scope actually allowed for more focus on this aspect of the program. As the US Program Director said:

One good thing, from my perspective was that we were able to develop the senior leadership curriculum, and to really dig-in on the relationships of the senior girls who had been involved for three years now. To watch what the Kenyan team built and delivered, and thinking how we might utilize that in the future, was a huge step forward for our organization.

It was clear from the data that *Educational Advancement* was also something HOH needed to adapt in their delivery to ensure participant needs were being met during the pandemic.

### Provision of Essential Supplies

Finally, the need for the *Provision of Essential Supplies* became immediately apparent as the pandemic led to most of the participants' parents losing their casual labor jobs. This meant that most parents were home without money. Most of the parents do not have savings and had little way to provide for their children. As Diana shared,

Due to the pandemic, everyone has been forced to stay home and when this happened many people lost their jobs and unemployment increased. There is a need for food and shelter, which are the main things. The food prices have also raised. However, there is still hope because HOH came at the right time to help people. They assist people by giving them food and other necessities. I am really grateful for their help.

Mila added, “What I have been needing is food, studies, and sanitary towels. I want to thank HOH because they have provided what I needed.”

The HOH program responded by organizing food/supply distributions within 2 weeks of the government shut-down. Since then, the HOH program staff has continued shopping for food stuffs and other essentials like sanitary pads at least twice a month to relieve the pressure from the parents and the families. In the words of the Kenyan Program Director:

Working with the coaches and mentors and with consultation and financial support from our U.S. partners, we have been able to redirect some funds that were budgeted for appreciating mentors. This money has been used to buy food supplies for the girls in the program whose families have been hardly hit by this pandemic. The food supplies have helped to cushion the girls and their families, from hunger, hustles of child labor and sexual exploitation in search of daily bread. This has greatly aided to keep our girls safe from sexual transmitted diseases, teenage pregnancies, child labor and sexual assaults and abuse.

The program implementors were ambitious and creative in developing plans for food distribution and in raising the funding for much of the initiative. In addition, the program leaders suggest that this distribution helped develop closer relationships with the participants' families, which will continue to build trust not only in these families, but also across the community.

In summation, *Love, Safety, and Support, Educational Advancement*, and *Provision of Essential Supplies* were the most salient participants needs. To meet those needs, HOH, at the direction of grassroots providers adapted both content and delivery of their SFD programming to provide for these needs during the COVID-19 pandemic.

### Impact on the Future of the Organization

Research Question 3 addressed how the program changes might affect the future of the organization. There were three main themes related to the future of the organization: (1) *Core Values*, (2) *Process Tensions*, (3) *Delivery*. Each of these is discussed below.

### Core Values

First, the HOH program response to changing participant needs highlighted the *Core Values* of the organization that will continue to inform its future. That is, the core value of loving and supporting young people was strongly reified through the experiences with COVID-19. As the Head Program Mentor and Head Basketball Coach discussed, “The main program interest when dealing with kids is the love, care, and support. When this is achieved any other challenges can be tackled.” This sentiment was affirmed in the bi-monthly conference calls where the entire program staff discussed that the actual content and specific delivery mechanisms of the HOH program were all flexible as long as they were directed toward loving and serving local young people.

### Process Tensions

Second, the organization's response to the participant needs highlighted a few *Process Tensions* within the organization. These were mainly related to discussions about which organizational members were responsible for what program aspects (e.g., design, implementation), and how funding and other resources would be distributed to the various stakeholders. As documented in the internal program documents, bi-monthly conference calls, prior to COVID-19, the program staff agreed to a set of organizational goals with a timeline and designated personnel. The drastic shift in participant needs coupled with government restrictions essentially negated the entire program plan that had been agreed upon.

This created some tension within the organization based on internal agreements about what activities each stakeholder would be responsible for conducting and contributing to the organizational goals. As reflected in an informal interview with the Head Mentor:

It's difficult because we want to do the best thing for the girls, but we are not able to receive the support from the organization as a whole because they cannot come here where we are. We cannot leave the girls with nothing, but we are not sure what to do. So that is troublesome.

The field notes from the U.S. Program Director echo this tension,

I am in uncharted waters. I feel that we should just release all obligations from the time COVID started, but I am not sure if that is the right way to go. It seems unreasonable to hold people accountable for activities they have NO WAY of carrying through. I think the best way forward is to start fresh with expectations from everyone.

According to the field notes, the organization essentially did that, collectively revamping the roles and responsibilities of the US Program Director, Kenyan Program Director, Head Basketball Coach, and Head Mentor according to the newly defined participant needs, and the activities that seemed reasonable and feasible to conduct at least for the remainder of 2020. The organization quickly moved forward at the direction of the program implementers.

### Program Delivery

Finally, the adaptations necessary to work through COVID-19 provided an opportunity to revisit and reconsider the future of HOH *Program Delivery*. For example, the Kenyan Program Director mentioned that prior to COVID-19 the program focus “has been solely school based and child/teen centered/oriented. So, COVID-19 has challenged our working/implementation model.” This presented a challenge, in terms of delivery because schools were closed. This also created an opportunity to reconsider delivery. In her words,

In future this calls for a more holistic approach, and includes parents and caregivers/guardians at the onset of the program implementation, especially at recruitment level. This makes follow-ups easier, and trust is built and enhanced during earlier stages of the program.

Because the program has been operating almost entirely through communication with the schools, the closing of schools created a massive communication gap between HOH and their participants, which can be addressed through those recommended changes.

Another challenge that the Kenyan Program Director expressed during the COVID-19 season was technology. While they had put together “plans and projections to conduct e-mentoring sessions with mentees,” they experienced “major setbacks due to the lack of necessary quotient and technology know-how of some mentors and mentees.” For future delivery of the HOH program she recommended increased investment “on building the capacities of the volunteers, and both mentors and coaches on matters of IT and social media.” Further she notes that it would be helpful to also empower the program participants on “responsible, disciplined, use and access to social media platforms.”

## Discussion

The COVID-19 pandemic represented a turning point both for individuals and organizations around the world (Giele and Elder, 1998). SFD programs also strongly felt this impact. While it may be to varying degrees, facility closures and sport and play activity cancellations around the globe changed the routines, social interactions, and overall daily norms of sport programs and sport participants on a global scale (Kelly et al., 2020). The results from this study highlight the impact this turning point had on the individual lives of grassroots SFD participants (RQ1). The participants not only identified positive takeaways and challenges from the fallout of COVID-19, particularly as schools were shut down and their activities restricted, but importantly provided insight on emergent participant needs (RQ2) and future directions for SFD programs (RQ3). They clearly articulated responses to that turning point, including short and long-term feelings and strategies for coping with the change. The impact of COVID-19 illuminated the core participant needs for *Love, Safety, and Security, Education*, and *Provision of Essential Supplies*. The focal case organization, Highway of Hope, responded in multiple ways, demonstrating flexibility in content, place, and delivery model. Consistent with Warner and Martin (2020) COVID-19 indeed created an opportunity for this sport organization to further justify their role in a society. It was clear that HOH has and can continue to impact lives, but must adjust, learn, and view the COVID-19 pandemic as an opportunity.

The framing of COVID-19 as both an individual and organizational turning point supports the use of life course theory for understanding individual's sport experiences. Clearly, these sport experiences are shaped and constrained by historical and social forces beyond their control, and exploring both the impact and the response can provide a richer understanding of the lived experiences of SFD participants around the globe.

Further, this study extends Warner and Martin (2020) by adding empirical data regarding the lived experiences of participants in a grassroots SFD organization in Kenya. To echo their charge, the results from this demonstrate “just how fundamental sport is to individuals' overall well-being, health, and everyday life.” The removal of sport from the participants in this organization had drastic impacts on their relationships with others, their daily activity routines, their *Socio-emotional* and *Physical Health*, and their *Sport-specific* skills. Prior to COVID-19, sport was clearly making a positive impact on their lives and the removal of sport was felt well-beyond the act of playing the game.

At an organizational level the results demonstrated the ability of grassroots SFD organization to rapidly identify and respond to emerging participant needs. This organizational response highlights both the tensions and the adaptability of a hybrid grassroots organizations (see Dixon and Svensson, 2019), and provides several points for discussion and learning in other contexts. First, it appears that the flexible nature of the organization allowed for meaningful and relatively rapid shifts in organizational programming and delivery. SFD scholars have suggested that top-down policies and restrictions from external stakeholders can limit the ability of grassroots organizations to respond to contextual needs (e.g., Schulenkorf, 2012; Svensson, 2017). In spite of minor organizational tensions that needed to be resolved, the organization did not appear to be restricted in its adaptations by such top-down pressures from external stakeholders. In fact, local leaders appeared to be empowered and confident in their ability to address the challenges in response to emergent participant needs. Thus, the actions of the program seem more in line with arguments suggesting that hybrid organizations, given the right conditions, can create new ways of responding to problems (e.g., Doherty et al., 2014; Dixon and Svensson, 2019). This organization seemed to pivot around the actions and advice of the program implementors who initiated changes in program design and delivery.

It appears that the organizational structure and values were flexible enough to adapt to the organizational changes, but it is not entirely clear what other factors led to the ability of the organization to adapt quickly. Previous scholars have suggested that trust, cultural sensitivity, and openminded organizational leadership are essential for maximizing the strengths of hybrid organizations (Smith et al., 2012; Doherty et al., 2014; Dixon and Svensson, 2019). The enduring involvement of both the Kenyan and U.S. partners over time worked to build such an organizational culture may have played a role. While this study supports the assertion that hybrid organizations can be highly adaptable, clearly there is room for additional empirical and theoretical advancement concerning the wider contextual contingencies that impact hybrid organizations' response to organizational turning points such as COVID-19.

The study was limited to the examination of one SFD organization, and the participant experiences of young women in a high school setting. Because the study was focused on life course theory and on understanding organizational responses to turning points, it was not focused specifically on women's experiences. However, it is possible that the life course experiences of these young women are gendered in nature (see Bruening and Dixon, 2008; Dixon et al., 2008) and may not apply to programs that serve boys and girls, or ones that serve primarily boys. Future research should examine the differences in experiences, interpretations, and impacts both of SFD, and of COVID-19 based on gender.

### Implications for SFD Organizations

The results from this study suggest that SFD organizations need to continue to focus on identifying the core needs of their constituents in program design, and also ensure that the programming meets those needs first, rather than the demands imposed by external stakeholders who may not understand or value the core needs of those participating in the programs (Giulianotti et al., 2016). External stakeholders also need to hear and value the voices of those delivering the program, such that they do not unnecessarily constrain the ability of grassroots providers to respond to needs, especially as those needs pivot in the face of turning points like COVID-19.

Second, this study points to the need for ongoing capacity building of grassroots providers for several reasons. First grassroots providers need to be able to respond to participant needs without “strong” dependency on external organizations. For example, in this organization, the primary program providers were able to initiate food distributions and secure a location for tutoring based on their own internal community networks. Then, the organization as a whole was able to support those needs and help broaden and sustain them. But the capability of the primary providers allowed for the most flexibility and rapid response from the organization. This could be a sign of the organizations' path toward sustainability, as the very definition of organizational sustainability according to Lindsey (2008, p. 283) is “the maintenance or expansion of sports development programs by the organization responsible for their delivery.” This is an example of how local leadership can and should be able to tackle challenges as they arise in their communities, not having to wait or rely on external involvement.

Building capacity of grassroots providers also enhances their capability to respond to a broader array of participant needs. For example, within this organization, basketball coaches were able to at least provide rudimentary tutoring and educational programming. If they had broader skills and/or training, they might have been able to provide even better educational components or more mental health support than a “listening ear.”

Third, the study points to the need for continuing to build SFD programming that enables and bolsters capacity and independence of the participants themselves. That is, some SFD organizations by the design of their programming, actually develop a dependency on the organization (Schulenkorf, 2010). The young women in this organization struggled with moving toward a place where they were coping and feeling a sense of hope and survival with or without the HOH programming. HOH and other SFD organizations need to consider ways encourage and equip participants to learn their own training skills and develop their own practical educational skills such that they could survive and thrive even in the absence of the organization.

### Theoretical Implications

Along with the practical implications, this work also offers some important theoretical insights related to life course theory and turning points (Giele and Elder, 1998; Bruening and Dixon, 2008). Specifically, turning points within a life course perspective are traditionally used to understand an individual's intentional decisions at specific points in time or instigated by a significant social event. This study highlights how a life course perspective can and should be used to better understand organizations and organizational responses to significant events. By utilizing a life course approach, the results demonstrate how HOH, an SDF hybrid organization, was able to adapt and respond to COVID-19, a significant social event, in a manner that could not have been predicted or designed. This expands life course theory in its usefulness for understanding not only intentional decision points, but also responses to events outside the organization's control.

The results also extend theory in the area of organizational hybridity, whereby the events created opportunities for the organizational partners to continually renegotiate partner roles, expectations, and organizational logics. The study illuminated the need for organizational hybridity to be viewed not as a static state to which organizations arrive, but as a continuously responsive and dynamic process (see also Battilana et al., 2014; Dixon and Svensson, 2019). Future work in the area of hybridity should continue to combine the concepts of life course theory and turning points to help inform when, where, and how hybrid organizations develop and morph into various organizational forms over time and in response to their environment.

### Conclusion

The clear story of this study is the central role that sport plays in the lives of participants in a grassroots setting. The role of sport in this context extends well-beyond the playing of a game, but instead anchors the participants' individual and social lives. The participants' lived experience of COVID-19 is that it took away sport from them, which impacted not only their sport skills, but also their physical and socio-emotional well-being. Therefore, it is imperative that grassroots organizations are prepared, adaptable, creative, and participant-centric such that they can seamlessly continue to carry out their operations in the face of challenges, turning them instead into opportunities for new ways of advancing sport as a positive impact in individuals and communities around the world.

## Data Availability Statement

The datasets presented in this article are not readily available because these are confidential data per IRB requirements, particularly because the organization is named in the study. Requests to access the datasets should be directed to Marlene A. Dixon, madixon@tamu.edu.

## Ethics Statement

The studies involving human participants were reviewed and approved by Texas A&M University IRB. Written informed consent from the participants' legal guardian/next of kin was not required to participate in this study in accordance with the national legislation and the institutional requirements.

## Author Contributions

MD conceptualized the study, helped with the design of data collection, analyzed, interpreted, and wrote much of the manuscript and was the lead author. AH helped with conceptualization and framing, data analysis, and also contributed to technical aspects of the manuscript. SW helped with conceptualization, framing, and writing the results. EO and DO helped with conceptualization, design of the case study, research questions/journal prompts, and also collected and analyzed the data. All authors contributed to the article and approved the submitted version.

## Conflict of Interest

The authors declare that the research was conducted in the absence of any commercial or financial relationships that could be construed as a potential conflict of interest.
